# A longitudinal study of exposure to fine particulate matter during pregnancy, small-for-gestational age births, and birthweight percentile for gestational age in a statewide birth cohort

**DOI:** 10.1186/s12940-021-00823-x

**Published:** 2022-01-11

**Authors:** Mercedes A. Bravo, Marie Lynn Miranda

**Affiliations:** 1 Duke Global Health Institute, Duke University, 310 Trent Drive, Durham, NC 27710 USA; 2 Children’s Environmental Health Initiative, University of Notre Dame, South Bend, IN USA; 3 Department of Applied and Computational Mathematics and Statistics, University of Notre Dame, South Bend, IN USA

**Keywords:** Small for gestational age, Birthweight percentile for gestational age, PM_2.5_, Air pollution, Longitudinal

## Abstract

**Background:**

Previous studies observed associations between prenatal exposure to fine particulate matter (≤ 2.5 μm; PM_2.5_) and small-for-gestational-age (SGA) birth and lower birthweight percentile for gestational age. Few, if any, studies examine prenatal air pollution exposure and these pregnancy outcomes in neonates born to the same women. Here, we assess whether prenatal exposure to ambient fine particulate matter (PM_2.5_) is associated with small-for-gestational-age (SGA) birth or birthweight percentile for gestational age in a longitudinal setting.

**Methods:**

Detailed birth record data were used to identify women who had singleton live births at least twice in North Carolina during 2002–2006 (*n* = 53,414 women, *n* = 109,929 births). Prenatal PM_2.5_ exposures were calculated using daily concentration estimates obtained from the US EPA Fused Air Quality Surface using Downscaling data archive. Associations between PM_2.5_ exposure and birthweight percentile and odds of SGA birth were calculated using linear and generalized mixed models, comparing successive pregnancies to the same woman. Odds ratios and associations were also estimated in models that did not account for siblings born to the same mother.

**Results:**

Among NHW women, pregnancy-long PM_2.5_ exposure was associated with SGA (OR: 1.11 [1.06, 1.18]) and lower birthweight percentile (− 0.46 [− 0.74, − 0.17]). Trimester-specific PM_2.5_ was also associated with SGA and lower birthweight percentile. Among NHB women, statistically significant within-woman associations between PM_2.5_, SGA, and birthweight percentile were not observed. However, in models that did not account for births to the same mother, statistically significant associations were observed between some PM_2.5_ exposure windows and higher odds of SGA and lower birthweight percentile among NHB women.

**Conclusions:**

Findings suggest that a woman is at greater risk of delivering an SGA or low birthweight percentile neonate when she has been exposed to higher PM_2.5_ levels. The within-woman comparison implemented here better controls for factors that may differ between women and potentially confound the relationship between PM_2.5_ exposure and pregnancy outcomes.

This adds to the evidence that PM_2.5_ exposure may be causally related to SGA and birthweight percentile, even at concentrations close to or below National Ambient Air Quality Standards.

**Supplementary Information:**

The online version contains supplementary material available at 10.1186/s12940-021-00823-x.

## Introduction

Birth outcomes are linked with adverse health outcomes over the life course [1, 2]. Small for gestational age (SGA) birth, defined as <10th percentile birthweight for gestational age, is associated with neonatal morbidity and mortality [3], childhood obesity [4], poorer academic outcomes [5], and lower socioeconomic status (SES) in adulthood [6]. Exposure to ambient air pollution during pregnancy may increase risk of lower birthweight percentile for gestational age. Fine particulate matter with an aerodynamic diameter *<* 2.5 μm (PM_2.5_) has been associated with adverse pregnancy outcomes, including SGA birth [7], but this body of work has several limitations.

First, most studies of PM_2.5_ exposure during pregnancy compare exposure and pregnancy outcomes in different women. It is uncertain whether the results are affected by individual characteristics, such as genetic factors [8] or social conditions [9] that vary considerably between women. That is, it is unclear whether observed associations reflect causal effects, as the women with the highest exposures may have other risk factors. To the best of our knowledge, there are no population-based studies of prenatal PM_2.5_ exposure and SGA or birthweight percentile for gestational age in the United States that compare successive pregnancy outcomes in the same mother.

Second, the few population-based studies of birth outcomes examining neonates born to the same women are concentrated in the northeastern US. In Connecticut [10, 11] and Rochester, NY [12], associations were observed between prenatal PM_2.5_ exposure and preterm birth among infants born to the same woman. However, the composition, seasonality, and overall concentration of PM_2.5_, which may contribute to health effects [13, 14], differ across the US [15]. States in the southern US also consistently have the highest prevalence of adverse pregnancy outcomes [16]. Thus, we should proceed with caution when generalizing associations between PM_2.5_ and pregnancy outcomes across US regions and populations.

Third, some studies rely on PM_2.5_ exposure estimates based on ambient monitors, which are predominantly located in urban and suburban areas [17]. These studies typically include only women within a certain distance of an active monitor; as a result, women in more rural areas are often excluded. More recent research has begun to address this by leveraging satellite data, land use regression, and air quality models to expand and diversify the sample for which exposures and health effects can be estimated [18]. Some results suggest that health effects estimated based on populations near and far from monitors differ from one another [19, 20], and more research is needed to understand the generalizability of relationships observed between PM_2.5_ exposure and pregnancy outcomes.

Using 5 years (2002-2006) of North Carolina Detailed Birth Records (NCDBR), we identify women who gave birth at least twice during this window. We estimate each woman’s exposure to ambient PM_2.5_ during pregnancy using daily concentrations estimated by the Community Multiscale Air Quality (CMAQ) model downscaler. Our objective is two-fold: (1) evaluate whether a woman is at greater risk of delivering a SGA infant when she has experienced elevated exposure to ambient PM_2.5_ during pregnancy compared to when she has not by comparing pregnancies in the same woman; and (2) evaluate whether a woman is at greater risk of delivering an infant with lower birthweight percentile for gestational age when she has experienced elevated exposure to ambient PM_2.5_ during pregnancy compared to when she has not.

## Methods

### Study design

This was a longitudinal study of a retrospective cohort of women who gave birth in North Carolina. We examined repeated pregnancies to the same women. The exposure of interest was ambient PM_2.5_ during pregnancy and outcomes of interest were birthweight percentile for gestational age and SGA births across successive pregnancies. Birthweight percentile for gestational age was calculated by comparing each infant’s recorded birth weight and gestational age in weeks to a sex-specific national reference distribution of birth weight for gestational age at delivery (21). Infants were designated as SGA if their birth weight was <10th percentile for their gestational age [21, 22].

### Study population

NCDBR were obtained from the State Center for Health Statistics for 2002–2006. The NCDBR includes all registered live births in the state during the study period (*n* = 604,757). Infant characteristics obtained from the birth records included clinical estimate of gestational age (weeks), infant sex, birth weight, birth order, congenital anomalies, plurality, and date of birth, among others. Maternal characteristics in the NCDBR included residential address at time of birth, age, marital status, education, race and ethnicity, tobacco use, parity, and the trimester in which prenatal care began, among others.

We street geocoded individual maternal residential addresses to the Census block level. Approximately 14% (*n* = 101,661) of births in the NCDBR could not be geocoded and were omitted from further analysis (remaining *n* = 521,715). We restricted to singleton live births without congenital anomalies, with birth weight > 400 g, parity < 4, a clinical estimate of gestation between 24 and 42 weeks, maternal age between 15 and 44 years (remaining *n* = 461,655). We restricted to births occurring to non-Hispanic Black (NHB) and non-Hispanic White (NHW) women due to small cell sizes for other races/ethnicities (remaining *n* = 393,012). We excluded births with missing covariate data (remaining *n* = 392,358). Similar restrictions have been applied in other studies of prenatal air pollution exposure and pregnancy outcomes [10, 23].

We also restricted to women who had weather (temperature) data available during their pregnancies (remaining *n* = 391,643).

The resulting reference population, which included women with one birth in the study period, consisted of birth records for 391,643 neonates born to 335,014 women. For the longitudinal (sibling) analysis, the reference population was restricted to women who gave birth at least twice during the study period. We excluded 215 births to 101 women for whom maternal race could not be determined because different maternal races were listed for different births. This resulted in a final longitudinal study population of 109,929 neonates born to 53,414 unique women, including 28,782 infants born to 13,797 NHB women and 81,147 infants born to 39,617 NHW women.

The characteristics of the final reference dataset (*n* = 391,643 neonates) and sibling dataset (*n* = 109,929 neonates) differed from those of the initial detailed birth record dataset consisting of 604,757 unique neonates born in North Carolina between January 1, 2002 and December 31, 2006 (Supplemental Material [SM], Table S1). Compared to neonates in the initial dataset, neonates in the final reference population and longitudinal study population had higher birthweight percentiles for gestational age and were less likely to be SGA. Smoking during pregnancy was more common in the reference and longitudinal study population compared to the initial dataset. SES proxies (e.g., educational attainment, marital status, median household income in the census tract of residence) were lowest in the initial dataset and highest in the longitudinal study population, suggesting that the longitudinal study population may consist of higher SES women. Racial composition also differed: initially, 23.0% of mothers were NHB, 58.1% were NHW, 14.7% were Hispanic, 2.68% were non-Hispanic Asian/Pacific Island, and 1.42% were non-Hispanic other, compared to a racial composition of 26.2% NHB and 73.8% NHW in the longitudinal study population.

### Exposure assessment

Daily 24-h averages of ambient PM_2.5_ concentrations from 2001 to 2006 were obtained from the publicly available Fused Air Quality Surface using Downscaling (FAQSD) files [24]. FAQSD files include estimates of daily 24-h average PM_2.5_ and 8-h maximum O_3_ concentrations at US census tract centroids. Inputs to the downscaler include monitoring data from the National Air Monitor Stations/State and Local Air Monitor Stations network and output from the Community Multiscale Air Quality (CMAQ) numerical model, specifically, 24-h PM_2.5_ concentrations at 12 × 12 km grid cells. The CMAQ model is a regional air quality model that estimates pollutant concentrations and deposition fluxes at local, regional, and continental scales. Using meteorological and emissions data, CMAQ simulates pollutant transformation, transport, and fate. Meteorological variables were estimated using 5th generation Mesoscale Model. The emissions inventory was based on the National Emissions Inventory and daily continuous emissions monitoring data for major point sources of nitrogen oxides. The downscaler uses monitoring data, gridded CMAQ output at 12 × 12 km grid cells, and regression models with additive and multiplicative bias coefficients that vary spatially and temporally to predict daily air pollution concentrations at census tract centroids. FAQSD data files are publicly available on the United States Environmental Protection Agency website. Further details on the downscaler methodology, results, and validation are available elsewhere [25–28].

Air pollution exposure during pregnancy was calculated based on census tract of residence at the time of each delivery, infant birth date, and length of gestation (weeks). We linked births to the census tract corresponding to each mother’s residence at the time of delivery to estimate PM_2.5_ exposure. The time of exposure for each birth was calculated using the date of birth and the weeks of gestation at delivery, as recorded in the NCDBR. Using 24-h average ambient PM_2.5_ concentrations, we calculated average exposure for the entire pregnancy and first (1-13 weeks), second (14-26 weeks), and third (27 weeks-birth) trimesters.

Daily observations of minimum, maximum, and average ambient temperature were obtained from weather stations in NC or within 50 km of the border in a neighboring state from the National Climatic Data Center [29]. Mothers were assigned temperature data based on the closest weather monitor within 50 km. Temperature exposures were calculated for the same exposure windows as PM_2.5_.

### Statistical analysis

Birthweight percentile for gestational age and SGA were the outcomes of interest. We used generalized linear mixed models with a random intercept on mother to estimate associations between trimester-specific and pregnancy-long PM_2.5_ exposure and odds of delivering an SGA neonate. Linear mixed models with a random intercept on mother were utilized to estimate associations between PM_2.5_ and birthweight percentile for gestational age.

Additionally, we fit “standard” models that were naïve to siblinghood. These included a logistic regression model for SGA and a linear regression model for birthweight percentile for gestational age, which were fit on both the longitudinal study population (only neonates born to mothers with ≥2 births during the study period) and the reference population (all neonates, including those born to mothers with only 1 birth during the study period).

Models adjusted for maternal characteristics, including education (less than high school, high school graduate, college graduate), age at delivery (15-19, 20-24, 25-29, 30-34, 35-39, 40-44 years), marital status, and smoking status; infant characteristics, including birth order (1, 2, 3, 4) and sex; trimester-specific and whole pregnancy average ambient temperature (°F); and median household income in the census tract of residence. Adjustment was made for these variables because of their potential to change between pregnancies and because they may be independent risk factors for lower birthweight [10].

Preliminary exploratory analysis revealed differential covariate distributions by race group (Table 1). Thus, we fit race-stratified models of SGA and birthweight percentile for gestational age regressed on PM_2.5_ and adjusting for individual-level factors, separately for trimester-specific and whole-pregnancy PM_2.5_ exposure.

**Table 1:** Characteristics at study entry for women residing in North Carolina who delivered at least two singleton neonates, 2002-2006

	Non-Hispanic black (*n* = 13,797) n (%)^a^	Non-Hispanic white (*n* = 39,617) n (%)^a^	*p*-value^b^
Infants born SGA	2940 (21.3)	2305 (5.82)	< 0.001
Mean birthweight percentile for gestational age	37.3 (26.5)	51.3 (27.9)	
Age of mother, years			< 0.001
< 20	4372 (31.7)	4993 (12.6)	
20-24	5333 (38.6)	9877 (25.0)	
25-29	2442 (17.7)	12,015 (30.3)	
30-34	1248 (9.05)	10,078 (25.4)	
35-39	373 (2.70)	2514 (6.32)	
40-44	31 (0.22)	130 (0.33)	
Educational level, years			< 0.001
No high school diploma	4308 (31.2)	5352 (13.5)	
High school diploma	7727 (56.0)	17,281 (43.6)	
College diploma	1762 (12.8)	16,894 (42.9)	
Not married	10,052 (72.9)	8072 (20.4)	< 0.001
Birth order			< 0.001
1	7865 (57.0)	27,584 (69.6)	
2	4092 (29.7)	8921 (22.5)	
3	1794 (13.0)	3053 (7.71)	
> 4	46 (0.33)	59 (0.15)	
Smoker	1412 (10.2)	5491 (13.9)	< 0.001
Median household income (census tract)	$35,239 (13,115)	$46,485 (16,621)	< 0.001

^a^The cell count and percentage are presented except in the cases of birthweight percentile for gestational age and median household income, where the mean and standard deviation are provided

^b^The chi-square test was used to test for differences by race group for categorical variables. The Kruskal-Wallis rank sum test was used birthweight percentile for gestational age and census tract-level median household income

### Sensitivity analysis

We conducted several sensitivity analyses. Specifically, we fit models that adjusted for average maximum temperature during each exposure period (e.g., 1st, 2nd, 3rd, trimester, whole pregnancy), instead of mean temperature during each exposure period. We also fit models in which we adjusted for trimester-specific and whole pregnancy ozone (O_3_) exposure (in addition to PM_2.5_). We also evaluated differences in the study population used in the main analysis versus the study population had we restricted to mothers who resided within 50 km of an active ambient PM_2.5_ monitor for > 2 births during the study period. This is the sample of women for which PM_2.5_ exposure estimates, and associations with pregnancy outcomes, are often estimated. Because of potential differences in the study population that could be included using available PM_2.5_ monitoring data vs. downscaler output, we estimated associations between birthweight percentile, SGA and: (1) PM_2.5_ exposures derived from monitoring data (for mothers residing within 50 km of an active PM_2.5_ monitor for at least 2 pregnancies during the study period); and (2) PM_2.5_ exposures derived from the downscaler (only in the subset of women residing within 50 km of PM_2.5_ monitor). This allows us to compare estimated associations between PM_2.5_ exposure, SGA, and birthweight percentile for gestational age for the full longitudinal study population versus the longitudinal study population for whom monitor-derived exposures are available. Lastly, we conducted analyses that excluded Caesarean section births.

This study was conducted in accordance with a human subjects research protocol approved by the Institutional Review Board at Rice University and the University of Notre Dame. All statistical analyses were performed in R v3.6.1 [30]. The glmer function in the lme4 package was used to fit generalized linear mixed models. The expression for likelihood of a mixed-effects model is an integral over the random effects space, which for models with a logit link was approximated with the Laplace approximation [31].

## Results

In the reference population (*n* = 391,643 neonates), SGA occurred among 9% of singleton live births during the study period. Prevalence of SGA differed considerably by race/ethnicity, with SGA occurring among 15.4 and 7.2% of births to NHB and NHW women, respectively. The average birthweight percentile was 39.0 for births to NHB women and 52.4 for births to NHW women.

The requirement of at least two births for inclusion in the longitudinal study population resulted in few women (< 1%) over age 40 years at time of study entry (Table 1). On average, NHB mothers tended be younger than NHW mothers: 38.6% of NHB mothers were age 20-24 years, compared to 25.0% of NHW mothers. Approximately 73 and 20% of NHB and NHW women were unmarried, respectively. Most women were nonsmokers, although a larger percentage of NHW women reported smoking during pregnancy compared to NHB women (13.9% vs. 10.2%, respectively). On average, NHW mothers had higher educational attainment than NHB mothers: 31.2% of NHB mothers did not graduate from high school, compared to 13.5% of NHW mothers, while 12.8% of NHB mothers were college graduates, compared to 42.9% of NHW mothers. At study entry, 21.3% of NHB women and 5.82% of NHW women had an SGA birth. Characteristics of the longitudinal study population at first and last birth are described in SM, Table S2. At their last birth, women were more likely to be older, married, have higher educational attainment, and have infants that had higher birthweight percentile for gestational age (SM, Table S2). NHB women were more likely to report smoking in their last birth, and both NHB and NHW women resided in census tracts with slightly higher median house income in the last birth compared to their first birth.

Mean exposure to PM_2.5_ in pregnancy was 13.7 μg/m^3^ and 13.5 μg/m^3^ for NHB and NHW mothers, respectively (Table 2). Among NHB women, the mean whole-pregnancy exposures for the first and last births in the study period were 13.6 μg/m^3^ and 13.8 μg/m^3^, respectively (*P* < 0.001). Among NHW women, the mean whole-pregnancy exposures for the first and last births in the study period were 13.4 μg/m^3^ and 13.6 μg/m^3^, respectively (*P* < 0.001).

**Table 2:** Exposure to PM_2.5_ and O_3_ and Ambient Temperature for the Longitudinal Study Population of Women Residing in North Carolina Who Delivered at Least 2 Singleton Neonates, 2002-2006

Exposure variable	First trimester		Second trimester		Third trimester		Whole pregnancy	
	Mean (IQR)	Min, Max	Mean (IQR)	Min, Max	Mean (IQR)	Min, Max	Mean (IQR)	Min, Max
Non-Hispanic black women (*n* = 13,797)								
PM_2.5_	13.6 (3.16)	7.32, 22.1	13.7 (3.26)	7.18, 22.4	13.7 (3.30)	5.64, 27.34	13.7 (1.89)	7.99, 19.1
O_3_	43.2 (17.4)	24.2, 70.3	43.5 (16.8)	24.2, 70.5	43.0 (17.5)	23.1, 77.2	43.2 (5.67)	30.0, 60.2
Temperature (°F)	60.3 (23.0)	30.4, 84.4	61.0 (23.0)	35.4, 83.5	60.9 (23.2)	31.8, 84.1	60.7 (7.80)	43.1, 76.0
Non-Hispanic white women (*n* = 39,617)								
PM_2.5_	13.5 (3.55)	6.59, 21.5	13.5 (3.39)	6.68, 22.2	13.6 (3.54)	6.59, 25.7	13.5 (1.94)	7.55, 18.6
O_3_	43.4 (16.7)	24.2, 71.1	43.5 (16.5)	24.2, 71.3	43.5 (16.5)	21.4, 73.5	43.5 (5.62)	28.7, 60.9
Temperature (°F)	59.7 (23.1)	24.7, 85.8	59.8 (22.5)	24.4, 83.6	60.2 (23.5)	22.6, 84.3	59.9 (7.62)	36.4, 78.1

Histograms showing differences in trimester-specific and pregnancy-long PM_2.5_ exposures between first and subsequent births for NHB and NHW women (SM, Fig. S1). A higher proportion of NHB women had smaller differences in PM_2.5_ exposure between pregnancies compared to NHW women, evidenced by the taller peak around 0 for NHB women, and the slightly taller tails for NHW women. The magnitude of within-woman differences was also smaller for pregnancy-long PM_2.5_ exposures compared to trimester-specific PM_2.5_ exposures, likely due to the shorter averaging interval and seasonal variations in PM_2.5_. Trimester-specific and pregnancy-long average PM_2.5_ and O_3_ exposures for first and last births are provided in SM, Table S3.

Birthweight percentile for gestational age differed more between women than between pregnancies of the same woman. For NHB birth weight percentile, approximately 71% of the variation occurred between NHB women, with the remaining 29% due to variation between pregnancies to the same NHB woman. Among NHW women, 77% of the variation in birthweight percentile occurred between women, with the remaining 23% due to variation between pregnancies to the same woman.

Most women, including 74.4% of NHB and 88.8% of NHW women delivered only non-SGA neonates, while small proportions (4.93 and 1.61% of NHB and NHW women, respectively) delivered only SGA neonates. There were 2854 NHB women and 3763 NHW women who delivered both SGA and non-SGA neonates.

Among NHW women, when longitudinal (“sibling”) models were used (i.e., models with a random intercept on mother), we observed the strongest evidence for an association of SGA with PM_2.5_ exposure during the whole pregnancy (Table 3). The odds of SGA birth increased by a factor of 1.11 (95% CI: 1.06, 1.18) per interquartile range (1.9 μg/m^3^) increase in PM_2.5_ exposure over the entire pregnancy (Table 3). First, second, and third trimester PM_2.5_ exposures were also associated with increased odds of SGA among NHW women. Older maternal age, smoking during pregnancy, and being unmarried at time of birth were associated with increased risk of SGA. Higher maternal educational attainment and higher birth order were associated with lower risk of SGA. Among NHW, second trimester, third trimester, and whole pregnancy PM_2.5_ exposures were also associated with statistically significant decrements in birthweight percentile for gestational age among NHW women (Table 4). The largest decrement, − 0.46 (95% confidence interval: − 0.74, − 0.17), was observed for PM_2.5_ exposure over the entire pregnancy.

**Table 3:** Odds Ratios by Exposure Period and Race for Small for Gestational Age (SGA) Births per Interquartile Range Increase in PM_2.5_ (1.9 μg/m^3^)^a^

	Longitudinal study population		Reference population
**Non-Hispanic Black women**	**SIBLING MODEL** **(*n*** **= 28,782 births to** ***n*** **= 13,797 women)**	**NAÏVE MODEL** **(*n*** **= 28,782 births)**	**NAÏVE MODEL** **(*n*** **= 105,262 births)**
1st trimester	1.01 (0.96, 1.06)	1.01 (0.97, 1.04)	1.01 (0.98, 1.02)
2nd trimester	1.02 (0.97, 1.07)	1.02 (0.98, 1.05)	1.02 (1.00, 1.03)
3rd trimester	1.05 (1.00, 1.10)	1.04 (1.01, 1.08)*	1.03 (1.01, 1.05)*
Whole pregnancy	1.04 (0.98, 1.11)	1.04 (1.00, 1.09)	1.03 (1.00, 1.05)
**Non-Hispanic White women**	**SIBLING MODEL** **(*n*** **= 81,147 births to** ***n*** **= 39,617women)**	**NAÏVE MODEL** **(*n*** **= 81,147 births)**	**NAÏVE MODEL** **(*n*** **= 286,381 births)**
1st trimester	1.08 (1.03, 1.12)*	1.07 (1.03, 1.10)*	1.03 (1.02, 1.05)*
2nd trimester	1.06 (1.02, 1.10)*	1.05 (1.02, 1.08)*	1.04 (1.02, 1.06)*
3rd trimester	1.06 (1.02, 1.11)*	1.06 (1.03, 1.09)*	1.04 (1.02, 1.05)*
Whole pregnancy	1.11 (1.06, 1.18)*	1.09 (1.05, 1.14)*	1.06 (1.04, 1.08)*

Abbreviations: *CI* confidence interval, *OR* odds ratio, *PM*_*2.5*_ particulate matter with aerodynamic diameter of < 2.5 μm

^a^ Models adjusted for birth order, infant sex, maternal age, maternal educational attainment, maternal smoking during pregnancy, maternal marital status, mean temperature during the corresponding exposure window, and median household income of the census tract of residence

*Indicates statistical significance at *p* < 0.05

**Table 4:** Associations by Exposure Period and Race for Birthweight Percentile for Gestational Age per Interquartile Range Increase in PM_2.5_ (1.9 μg/m^3^)^a^

	Longitudinal study population		Reference population
**Non-Hispanic Black women**	**SIBLING MODEL** **(*n*** **= 28,782 births to** ***n*** **= 13,797 women)**	**NAÏVE MODEL** **(*n*** **= 28,782 births)**	**NAÏVE MODEL** **(*n*** **= 105,262 births)**
1st trimester	0.094 (−0.26, 0.45)	− 0.035 (− 0.39, 0.31)	−0.05 (− 0.28, 0.086)
2nd trimester	− 0.011 (− 0.35, 0.33)	− 0.12 (− 0.45, 0.21)	−0.29 (− 0.46, − 0.11)*
3rd trimester	−0.19 (− 0.49, 0.18)	−0.37 (− 0.69, − 0.042)*	−0.21 (− 0.38, − 0.036)*
Whole pregnancy	−0.098 (− 0.57, 0.38)	−0.35 (− 0.77, 0.072)	−0.35 (− 0.57, − 0.13)*
**Non-Hispanic white women**	**SIBLING MODEL** **(*n*** **= 81,147 births to** ***n*** **= 39,617women)**	**NAÏVE MODEL** **(*n*** **= 81,147 births)**	**NAÏVE MODEL** **(*n*** **= 286,381 births)**
1st trimester	− 0.038 (− 0.25, 0.17)	− 0.20 (− 0.40, 0.01)	−0.19 (− 0.30, − 0.082)*
2nd trimester	**−0.34 (− 0.55, − 0.14)**	−0.38 (− 0.58, − 0.17)*	−0.34 (− 0.44, − 0.23)*
3rd trimester	**−0.31 (− 0.51, − 0.11)**	−0.45 (− 0.65, − 0.25)*	−0.31 (− 0.41, − 0.21)*
Whole pregnancy	**−0.46 (− 0.74, − 0.17)**	−0.54 (− 0.80, − 0.29)*	−0.43 (− 0.56, − 0.30)*

Abbreviations: *PM*_*2.5*_ particulate matter with aerodynamic diameter of < 2.5 μm

^a^ Models adjusted for birth order, infant sex, maternal age, maternal educational attainment, maternal smoking during pregnancy, maternal marital status, mean temperature during the corresponding exposure window, and median household income of the census tract of residence

*Indicates statistical significance at *p* < 0.05

Statistically significant associations were not observed between SGA and PM_2.5_ among NHB women for any of the exposure windows examined (Table 3). There were also no statistically significant associations between birthweight percentile for gestational age and PM_2.5_ exposure among NHB women (Table 4). As with NHW women, older maternal age, smoking during pregnancy, and being unmarried at time of birth were associated with increased risk of SGA. Higher maternal educational attainment and higher birth order were associated with lower risk of SGA.

Models that did not account for births occurring to the same woman were fit on two different datasets: (1) the longitudinal study population (i.e., the sibling dataset); and (2) the reference population.

In models naïve to siblinghood, among NHW women, statistically significant associations were observed between PM_2.5_ and increased odds of SGA for all exposure windows, in both the sibling and reference datasets (Table 3). The largest odds ratio was consistently for pregnancy-long PM_2.5_ exposure. Statistically significant associations were also observed between PM_2.5_ and lower birthweight percentile for gestational age for all exposure windows except first trimester exposure in the longitudinal study population (Table 4). The largest association was observed for full pregnancy PM_2.5_ exposure.

Among NHB women, in models naïve to siblinghood, third trimester PM_2.5_ exposure was associated with increased odds of SGA in both the longitudinal study population and the reference population (Table 3). Third trimester PM_2.5_ exposure was also associated with statistically significant decrements in birthweight percentile for gestational age among NHB mothers in the longitudinal study population (Table 4). Second trimester, third trimester, and whole pregnancy PM_2.5_ exposure were associated with statistically significant decrements in birthweight percentile for gestational age among NHB mothers in the reference dataset.

We conducted several sensitivity analyses. We checked linearity of associations by smoothing trimester-specific and pregnancy-long PM_2.5_ exposures for NHB and NHW mothers. Plots of smoothed effects are provided in SM, Figs. S2 and S3. Using O_3_ exposure estimates obtained from FAQSD data, we fit multipollutant models that adjusted for O_3_ and PM_2.5._ Correlations between PM_2.5_ and O_3_ for trimester-specific exposure estimates ranged from 0.62-0.64 and 0.60-0.63 for NHB and NHW, respectively. Correlations between pregnancy long PM_2.5_ and O_3_ exposure estimates were 0.41 for NHB and 0.34 for NHW. Associations observed between PM_2.5_ exposure, SGA, and birthweight percentile for gestational age were robust to separate adjustments for O_3_ (SM, Table S4) and ambient maximum temperature instead of mean temperature. Variance inflation factors (VIF) in models adjusting for both PM_2.5_ and O_3_ ranged from 1.33-2.98 in models for NHB women; VIF ranged from 1.42-2.81 in models for NHW women. VIFs in excess of 5 are generally a cause for concern [32, 33] and some researchers have argued for use of a more conservative threshold of 2.5 [34].

At time of study entry, SGA prevalence was higher for NHB mothers in the full longitudinal study population than for NHB mothers in the longitudinal study subsample that only included mothers with monitor-derived PM_2.5_ exposures (SM, Table S5). NHB mothers in the full longitudinal study population were, on average, less educated, more likely to be unmarried, and in census tracts with lower median household income compared to the subset of NHB mothers with monitor-derived PM_2.5_ exposure estimates. Among NHW, prevalence of SGA at time of study entry was lower in the full longitudinal study population compared to NHW mothers in the longitudinal study subsample that only included mothers with monitor-derived PM_2.5_ exposures. Compared to NHW mothers with monitor-derived PM_2.5_ exposures, NHW mothers in the full longitudinal study population were less educated, more likely to be unmarried, more likely to report smoking during pregnancy, and in census tracts with lower median household income.

Odds ratios and associations estimated using monitor- and downscaler-derived exposure estimates only for mothers with monitoring data were generally consistent with those reported in the main analysis (SM, Tables S5-S6). Odds ratios and associations estimated using all available downscaler data (main analysis) and monitoring data only were similar (sensitivity analysis), although associations with monitoring data were less likely to be statistically significant. This may be attributed to the reduction in sample size resulting from restriction only to mothers with PM_2.5_ monitoring data for two or more births. These findings suggest that the downscaler does a reasonable job of estimating air pollution exposure, relative to monitoring data. It also suggests that, at least in this case, monitor-derived exposure estimates may be generalizable to populations for whom monitoring data are not available.

Results of analyses that excluded Caesarean births were consistent with those reported in the main analysis, which included births by Caesarean section.

## Discussion

Using 5 years of geocoded NCDBR in which maternal siblings were identified, we evaluated whether a woman is at greater risk of delivering a SGA infant, or a lower birthweight percentile infant, when she has experienced elevated exposure to ambient PM_2.5_ during pregnancy. Although there are several studies that examine PM_2.5_ and within-mother associations with preterm birth [10–12], to the best of our knowledge, this is one of the first – perhaps the first – study to examine prenatal PM_2.5_ exposure and birthweight percentile-related outcomes in neonates born to the same mother.

In the longitudinal study population, exposure to PM_2.5_ during pregnancy was associated with higher odds of SGA among NHW mothers for all trimesters. The direction and magnitude of the associations observed in the longitudinal study population using the sibling models were generally consistent with those observed for the naïve models fit to the longitudinal study population and the reference population. Similarly, in sibling models, PM_2.5_ was associated with lower birthweight percentile for gestational age among NHW mothers in the longitudinal study population for second and third trimester and whole pregnancy PM_2.5_ exposure. This was consistent with findings from the naïve models.

Among NHB mothers, we did not observe statistically significant associations between PM_2.5_ and odds of SGA or birthweight percentile in sibling models. However, we did observe statistically significant associations between 3rd trimester PM_2.5_ exposure and odds of SGA in naïve models fit to the longitudinal study population and reference populations. Similarly, we also observed statistically significant associations between PM_2.5_ exposure and birthweight percentile for gestational age in naïve models fit to the longitudinal study population for 3rd trimester PM_2.5_ exposure, and in the reference population for 2nd trimester, 3rd trimester, and whole pregnancy PM_2.5_ exposure. The lack of statistical significance between PM_2.5_ and pregnancy outcomes among NHB mothers may be due to sample size. It is also possible that PM_2.5_ exposure differentials in pregnancies to the same mother were not large enough to provide a contrast in exposure sufficient to detect an association with pregnancy outcomes. In the analysis dataset, NHB women had, on average, smaller within-woman PM_2.5_ exposure differences in successive pregnancies compared to NHW women (e.g., SM, Fig. S1). This lower contrast may make it more difficult to detect within-woman associations between exposure and outcomes.

In utero, rapid growth and weight gain for fetus typically commences around week 30-31 [35]. Since much of a fetus’ weight gain occurs in the third trimester, air pollution exposure in the 3rd trimester may be more relevant to outcomes such as birthweight percentile for gestational age. At least one other study employing a similar design but examining preterm birth instead of SGA and birthweight percentile for gestational age also observed variability in most salient exposure windows for different racial/ethnic groups, including NHB and NHW [10]. Additionally, review articles examining the literature on air pollution exposure and pregnancy outcomes conclude that there is not yet consensus as to which trimester is most strongly associated with adverse pregnancy outcomes. The association between air pollution and birth outcome may depend on the air pollutant being examined, additional exposures experienced pre- and during pregnancy, and the birth outcome of interest (e.g., preterm birth versus SGA) [36, 37]. More research to further investigate this is needed.

Multiple studies report associations between PM_2.5_ exposure and SGA, although these studies compare exposures and pregnancy outcomes in different women. In NC, Gray et al. (2014) used FAQSD data to estimate exposures during pregnancy and associations with SGA and birthweight in grams [23]. An interquartile range (IQR) increase in pregnancy-long maternal PM_2.5_ exposure (2.0 μg/m^3^) was associated with increased odds of SGA birth (odds ratio: 1.03 [95% CI: 1.02, 1.05]). An IQR increase in pregnancy-long PM_2.5_ exposure was associated with a 3.1 g (95% CI: 3.0 to 3.2) reduction in birthweight [23].

In contrast, another NC-based study did not observe an association between prenatal PM_2.5_ and SGA. In adjusted single-pollutant models, Vinikoor-Imler observed associations close to the null or showing a possible reduction in risk of SGA associated with elevated PM_2.5_ exposure [38]. This study was also based in NC and used FAQSD data to estimate exposures but used a subset of the years (2003-2005) used here.

This study has several limitations. We generate PM_2.5_ exposure estimates from FAQSD data. FAQSD performance with respect to monitoring data has been evaluated previously [17, 20], but model performance cannot be assessed in areas without monitoring data. Differences between FAQSD-derived exposure estimates and monitor-derived exposure estimates may be greater in areas where there is less monitoring data available to use as inputs to the downscaler (i.e., more rural areas). Nonetheless, output from the downscaler has been used in multiple studies of air pollution exposure [17] and health [20, 23, 38]. We do not account for long-term trends in air pollution because our modeling approach relies on within-woman differences in air pollution exposure to detect an association, if any, with birth outcomes. If women had similar or the same air pollution exposure levels across multiple births, we would be unable to estimate an association between exposure and outcome because there would be no difference or contrast in the exposure levels. While we smoothed air pollution exposures as a sensitivity analysis, exploring and further characterizing potential non-linearities in associations between PM_2.5_ exposures and pregnancy outcomes is an important avenue for future research. Future work should also explore the role of O_3_ and SGA and birthweight percentile for gestational age in maternal siblings, as results from the multipollutant model are suggestive of an association between O_3_ exposure and these pregnancy outcomes, for some exposure windows (e.g., SM Table S4).

We examine SGA and birthweight percentile for gestational age outcomes within women. It has been argued that birthweight may not be an important outcome [39]. Nonetheless, our analysis of the continuous outcome birthweight percentile for gestational age allows us to understand whether, among the range of birthweight percentiles, women are more likely to have reductions in birthweight percentile when exposed to PM_2.5_.

Despite limitations, this study has important strengths. First, we compare PM_2.5_ exposure and pregnancy outcomes in successive pregnancies to the same women in NC. To the best of our knowledge, this is the first study to examine SGA and birthweight percentile for gestational age in neonates born to the same mother. Second, it is located in the American South, where the prevalence of adverse pregnancy outcomes (e.g., preterm birth, low birthweight) is higher [40]. Of related concern, seasonality, total concentration, and chemical composition of PM_2.5_ in NC and the southeastern US more generally differs from PM_2.5_ seasonality, total concentration, and chemical composition in other regions of the US [15]. Third, we estimate PM_2.5_ exposure and associations with SGA for mothers that do not reside near ambient monitors.

## Conclusion

These results add to the existing body of literature finding that prenatal air pollution exposure affects pregnancy outcomes, including SGA and birthweight percentile for gestational age. Specifically, the within-woman comparison utilized here better controls for social, genetic, and other factors that may differ between women and potentially confound the relationship between PM_2.5_, SGA and birthweight percentile. Thus, these findings suggest a possible causal relationship between PM_2.5_ exposure and pregnancy outcomes. This work also demonstrates that prenatal exposure to relatively low levels of PM_2.5_ is related to SGA and birthweight percentile, which is relevant to promulgation of public health-protective air quality standards.

## Supplementary Information

**Additional file 1: Supplemental material.** A Longitudinal Study of Exposure to Fine Particulate Matter during Pregnancy, Small-for-Gestational Age Births, and Birthweight Percentile for Gestational Age in a Statewide Birth Cohort. Tables and figures describing the changes in characteristics of the longitudinal study population between first and last birth.

## Data Availability

The air pollution datasets used and/or analysed during the current study are available from the corresponding author on request. Geocoded, long-form birth data are governed by an institutional review board and data use agreement with the North Carolina Department of Vital Statistics and are not publicly available.

## Abbreviations

CMAQ: Community Multiscale Air Quality
FAQSD: Fused Air Quality Surfaces using Downscaling
NC: North Carolina
NCDBR: North Carolina Detailed Birth Records
NHB: Non-Hispanic Black
NHW: Non-Hispanic White
PM_2.5_: Particulate matter with an aerodynamic diameter < 2.5 μm
SGA: Small for gestational age
