# Supplementary material for: A longitudinal study of exposure to fine particulate matter during pregnancy, small-for-gestational age births, and birthweight percentile for gestational age in a statewide birth cohort
Source: Environ Health. 2022 Jan 11;21:9. doi: 10.1186/s12940-021-00823-x (PMC8751317; doi:10.1186/s12940-021-00823-x)
Supplement: Supplementary file 1 — Additional file 1: Supplemental material. A Longitudinal Study of Exposure to Fine Particulate Matter during Pregnancy, Small-for-Gestational Age Births, and Birthweight Percentile for Gestational Age in a Statewide Birth Cohort. Tables and figures describing the changes in characteristics of the longitudinal study population between first and last birth. [file 12940_2021_823_MOESM1_ESM.docx]

**SUPPLEMENTAL MATERIAL**

A Longitudinal Study of Exposure to Fine Particulate Matter during Pregnancy, Small-for-Gestational Age Births, and Birthweight Percentile for Gestational Age in a Statewide Birth Cohort

Mercedes Bravo,^1,2,^ Marie Lynn Miranda^2,3^

^1^ Duke Global Health Institute, Duke University, Durham, NC, USA.

^2^ Children’s Environmental Health Initiative, University of Notre Dame, South Bend, IN, USA.

^3^ Department of Applied and Computational Mathematics and Statistics, University of Notre Dame, South Bend, IN, USA.

**Changes in characteristics of the longitudinal study population between first and last birth**

For most women, the first and last births during the study period correspond to the first and second birth during the study period, as only 8.27% of NHB mothers and 4.67% of NHW mothers had more than two births during the study period. There were no women with more than four births during the study period. Over 70% of NHB and NHW women remained in the same 5-year age category between their first and last birth in the study period. There were 701 (5.08%) NHB women and 2,119 (5.34%) NHW women with changes in marital status, predominantly changing from unmarried to married. Most women did not change smoking status between their first and last pregnancy, including 95.2% of NHB women and 96.4% of NHW women. Approximately 27.7% of NHB women and 18.6% of NHW women changed census tract of residence between their first and last births in the study period. Although 1,002 (7.26%) NHB women changed educational categories during the study period, the reported number of years of education anomalously decreased among 206 women. Similarly, among NHW, 1,431 (3.61%) women changed educational categories between first and last birth, with the reported number of years of education anomalously decreasing among 393 women.

**SM TABLES**

| **SM Table S1. Comparison of characteristics of the initial North Carolina detailed birth records versus the final analysis dataset(s)** | | | |
| --- | --- | --- | --- |
|  | Initial NCDBR dataset  (n=604,757)  N (%)^a^ | Final reference dataset  (n=391,643)  N (%)^a^ | Final sibling dataset  (n=109,929)  N (%)^a^ |
| **Child characteristics** | | | |
| Geocoded at time of birth | 521,715 (86.3) | 391,643 (100.0) | 109,929 (100.0) |
| Birthweight percentile for gestational age, mean | 47.2 (28.4) | 48.8 (28.4) | 49.7 (28.2) |
| Small for gestational age | 62,921 (10.4) | 36,859 (9.41) | 9,451 (8.60) |
| Male sex | 309,498 (51.2) | 200,411 (51.2) | 56,358 (51.3) |
| **Maternal characteristics**^c^ | | | |
| Race/ethnicity |  |  |  |
| NHB | 139,149 (23.0) | 105,262 (26.9) | 28,782 (26.2) |
| NHW | 351,119 (58.1) | 286,381 (73.1) | 81,147 (73.8) |
| Hispanic | 89,032 (14.7) | 0 | 0 |
| Non-Hispanic Asian/Pacific Islander | 16,207 (2.68) | 0 | 0 |
| Non-Hispanic other | 8,610 (1.42) | 0 | 0 |
| Reported smoking during pregnancy | 73,914 (12.2) | 52,879 (13.5) | 14,752 (13.4) |
| Age at time of child’s birth (years) |  |  |  |
| 15-19 | 68,492 (11.3) | 42,758 (10.9) | 12,834 (11.7) |
| 20-24 | 162,165 (26.8) | 101,758 (26.0) | 31,779 (28.9) |
| 25-29 | 162,682 (26.9) | 104,973 (26.8) | 29,068 (26.4) |
| 30-34 | 136,788 (22.6) | 93,253 (23.8) | 25,801 (23.5) |
| 35-39 | 61,226 (10.1) | 41,607 (10.6) | 9,462 (8.61) |
| 40-44 | 11,528 (1.91) | 7,294 (1.86) | 985 (0.90) |
| Educational attainment |  |  |  |
| Less than high school | 138,224 (22.9) | 55,723 (14.2) | 18,291 (16.6) |
| High school diploma | 306,598 (50.7) | 211,332 (54.0) | 53,039 (48.2) |
| College diploma or higher | 158,844 (26.3) | 124,588 (31.8) | 38,599 (35.1) |
| Unmarried at time of birth | 223,282 (36.9) | 129,916 (33.2) | 34,512 (31.4) |
| **Neighborhood characteristics** | | | |
| Median household income ($) | 42,423 (15,662) | 43,506 (15,963) | 43,791 (16,851) |
| ^a^The cell count and percent are presented except in the case of birth outcomes and neighborhood characteristics, where the mean is given as indicated next to the variable name.  ^b^ Maternal variables are based on reported maternal characteristics at time of the child’s birth. | | | |

| **Table S2. Characteristics at first and last birth for women residing in North Carolina who delivered at least two singleton neonates, 2002-2006** ^a,b^ | | | | |
| --- | --- | --- | --- | --- |
|  | Non-Hispanic Black  (n=13,797)  n (%) | | Non-Hispanic White  (n=39,617)  n (%) | |
|  | First birth | Last birth | First birth | Last birth |
| Infants born SGA | 2,940 (21.3) | 1,857 (13.5) | 2,305 (5.82) | 2,064 (5.21) |
| Mean birthweight percentile for gestational age | 37.3 (26.5) | 40.3 (26.8) | 51.3 (27.9) | 55.8 (27.5) |
| Age of mother, years |  |  |  |  |
| < 20 | 4,372 (31.7) | 1,469 (10.6) | 4,993 (12.6) | 1,407 (3.55) |
| 20-24 | 5,333 (38.6) | 6,311 (45.7) | 9,877 (25.0) | 8,996 (22.7) |
| 25-29 | 2,442 (17.7) | 3,498 (25.3) | 12,015 (30.3) | 10,484 (26.4) |
| 30-34 | 1,248 (9.05) | 1,696 (12.3) | 10,078 (25.4) | 12,310 (31.1) |
| 35-39 | 373 (2.70) | 706 (5.12) | 2,514 (6.32) | 5,720 (14.4) |
| 40-44 | 31 (0.22) | 117 (0.85) | 130 (0.33) | 700 (1.77) |
| Educational level, years |  |  |  |  |
| No high school diploma | 4,308 (31.2) | 3,347 (24.3) | 5,352 (13.5) | 4,353 (11.0) |
| High school diploma | 7,727 (56.0) | 8,553 (62.0) | 17,281 (43.6) | 18,078 (45.6) |
| College diploma | 1,762 (12.8) | 1,897 (13.7) | 16,894 (42.9) | 17,186 (43.4) |
| Not married | 10,052 (72.9) | 9,134 (66.2) | 8,072 (20.4) | 5,911 (14.9) |
| Birth order |  |  |  |  |
| 1 | 7,865 (57.0) | 0 (0) | 27,584 (69.6) | 0 (0) |
| 2 | 4,092 (29.7) | 7,069 (51.2) | 8,921 (22.5) | 26,047 (65.7) |
| 3 | 1,794 (13.0) | 4,448 (32.2) | 3,053 (7.71) | 9,993 (25.2) |
| > 4 | 46 (0.33) | 2,249 (16.3) | 59 (0.15) | 3,500 (8.83) |
| Smoker | 1,412 (10.2) | 1,780 (12.9) | 5,491 (13.9) | 5,514 (13.9) |
| Median household income (census tract) | $35,239 (13,115) | $35,650  (13,499) | $46,485 (16,621) | $47,129  (17,274) |
| ^a^The cell count and percentage are presented except in the cases of birthweight percentile for gestational age and median household income, where the mean and standard deviation are provided.  ^b^ Note that first and last birth correspond to first and last birth during the study period, and may not be the woman’s first and last birth overall (outside of the study period). Additionally, last birth corresponds to the second birth for most women, but a small percentage of women had more than 2 births during the study period. | | | | |

| **Table S3. Exposure to PM_2.5_ and O_3_ for the Longitudinal Study Population of Women Residing in North Carolina Who Delivered at Least 2 Singleton Neonates, 2002-2006** | | | | | | | | |
| --- | --- | --- | --- | --- | --- | --- | --- | --- |
| Exposure variable | **First trimester mean (IQR)** | | **Second trimester**  **mean (IQR)** | | **Third trimester mean (IQR)** | | **Whole pregnancy mean (IQR)** | |
| Pregnancy number | First | Last | First | Last | First | Last | First | Last |
| Non-Hispanic Black women (n=13,797) | | | | | | | | |
| PM_2.5_ | 13.8 (3.37) | 13.4 (3.00) | 13.5 (2.85) | 13.9 (3.86) | 13.4 (2.82) | 13.9 (3.90) | 13.6 (1.81) | 13.8 (1.96) |
| O_3_ | 44.1 (18.1) | 42.5 (17.2) | 43.1 (16.7) | 44.0 (16.8) | 42.6 (18.0) | 43.5 (16.9) | 43.3 (5.60) | 43.3 (5.77) |
| Non-Hispanic White women (n=39,617) | | | | | | | | |
| PM_2.5_ | 13.7 (3.77) | 13.3 (3.34) | 13.3 (2.99) | 13.7 (3.86) | 13.3 (3.02) | 13.8 (4.11) | 13.4 (1.86) | 13.6 (2.04) |
| O_3_ | 44.5 (17.7) | 42.4 (16.1) | 43.2 (16.2) | 43.9 (16.7) | 43.2 (16.9) | 44.0 (16.1) | 43.6 (5.68) | 43.4 (5.66) |

| **SM Table S4. Associations by Exposure Period and Race for SGA and Birthweight Percentile for Gestational Age per Interquartile Range Increase in PM_2.5_ (1.9 μg/m^3^), adjusting for ozone** | | | | |
| --- | --- | --- | --- | --- |
| **SIBLING MODEL: Small for gestational age (SGA) birth** | | | | |
|  | **PM_2.5_** | | **Ozone** | |
|  | **Non-Hispanic Black women**  **(n= 28,782 births to n= 13,797 women)** | **Non-Hispanic White women**  **(n=81,147 births to n= 39,617 women)** | **Non-Hispanic Black women**  **(n= 28,782 births to n= 13,797 women)** | **Non-Hispanic White women**  **(n=81,147 births to n= 39,617 women)** |
| 1^st^ trimester | 1.01 (0.95, 1.08) | **1.08 (1.02, 1.14)*** | 1.03 (0.98, 1.07) | **1.06 (1.03, 1.10)*** |
| 2^nd^ trimester | 1.05 (0.99, 1.11) | **1.07 (1.01, 1.13)*** | 1.03 (0.98, 1.07) | 1.02 (0.99, 1.06) |
| 3^rd^ trimester | 1.05 (0.98, 1.11) | **1.08 (1.02, 1.14)*** | 0.97 (0.94, 1.02) | 0.99 (0.96, 1.04) |
| Whole pregnancy | 1.07 (0.99, 1.16) | **1.11 (1.03, 1.19)*** | 1.02 (0.94, 1.11) | **1.11 (1.03, 1.180)*** |
| **SIBLING MODEL: Birthweight percentile for gestational age** | | | | |
|  | **PM_2.5_** | | **Ozone** | |
|  | **Non-Hispanic Black women**  **(n= 28,782 births to n= 13,797 women)** | **Non-Hispanic White women**  **(n=81,147 births to n= 39,617 women)** | **Non-Hispanic Black women**  **(n= 28,782 births to n= 13,797 women)** | **Non-Hispanic White women**  **(n=81,147 births to n= 39,617 women)** |
| 1^st^ trimester | 0.093 (-0.33, 0.52) | 0.035 (-0.21, 0.28) | -0.068 (-0.33, 0.20) | **-0.16 (-0.32, -0.010)*** |
| 2^nd^ trimester | -0.11 (-0.51, 0.30) | **-0.32 (-0.56, -0.081)*** | -0.25 (-0.52, 0.015) | -0.12 (-0.27, 0.034) |
| 3^rd^ trimester | -0.28 (-0.68, 0.12) | **-0.33 (-0.56, -0.091)*** | 0.076 (-0.19, 0.33) | 0.03 (-0.12, 0.18) |
| Whole pregnancy | -0.27 (-0.84, 0.31) | **-0.35 (-0.68, -0.017)*** | -0.37 (-0.95, 0.21) | **-0.37 (-0.69, -0.051)*** |

| **SM Table S5. Comparison of characteristics at study entry of births in the sibling sample versus births in the reference population** | | | | | |
| --- | --- | --- | --- | --- | --- |
|  | **Non-Hispanic Black** | | **Non-Hispanic White** | | |
|  | Full sibling sample, at time of study entry^a^  (n=13,797)  n (%) | Sibling sample with monitor-derived exposures, at time of study entry^a^  (n=9,675)  n (%) | | Full sibling sample, at time of study entry ^a^ (n=39,617)  n (%) | Sibling sample with monitor-derived exposures, at time of study entry^a^  (n=25,953)  n (%) |
| Infants born SGA | 2,940 (21.3) | 1,591 (16.4) | | 2,305 (5.82) | 1,903 (7.33) |
| Mean (SD) birthweight percentile for gestational age | 37.3 (26.5) | 37.7 (26.6) | | 51.3 (27.9) | 51.4 (27.9) |
| Age of mother, years |  |  | |  |  |
| < 20 | 4,372 (31.7) | 2,939 (30.4) | | 4,993 (12.6) | 2,906 (11.2) |
| 20-24 | 5,333 (38.6) | 3,646 (37.7) | | 9,877 (25.0) | 5,759 (22.2) |
| 25-29 | 2,442 (17.7) | 1,788 (18.5) | | 12,015 (30.3) | 7,912 (30.5) |
| 30-34 | 1,248 (9.05) | 988 (10.2) | | 10,078 (25.4) | 7,379 (28.4) |
| 35-39 | 373 (2.70) | 281 (2.90) | | 2,514 (6.32) | 1,893 (7.29) |
| 40-44 | 31 (0.22) | 23 (0.24) | | 130 (0.33) | 104 (0.40) |
| Educational level, years |  |  | |  |  |
| No high school diploma | 4,308 (31.2) | 2,880 (29.8) | | 5,352 (13.5) | 3,103 (12.0) |
| High school diploma | 7,727 (56.0) | 5,355 (55.4) | | 17,281 (43.6) | 10,434 (40.2) |
| College diploma | 1,762 (12.8) | 1,440 (14.9) | | 16,894 (42.9) | 12,416 (47.9) |
| Not married | 10,052 (72.9) | 6,859 (70.9) | | 8,072 (20.4) | 4,830 (18.6) |
| Birth order |  |  | |  |  |
| 1 | 7,865 (57.0) | 5,572 (57.6) | | 27,584 (69.6) | 18,249 (70.3) |
| 2 | 4,092 (29.7) | 2,847 (29.4) | | 8,921 (22.5) | 5,717 (22.0) |
| 3 | 1,794 (13.0) | 1,227 (12.7) | | 3,053 (7.71) | 1,952 (7.52) |
| > 4 | 46 (0.33) | 29 (0.30) | | 59 (0.15) | 35 (0.13) |
| Smoker | 1,412 (10.2) | 976 (10.1) | | 5,491 (13.9) | 3,212 (12.4) |
| Median household income (census tract) | $35,239 ($13,115) | $36,457 ($13,990) | | $46,485 ($16,621) | $49,586 ($17,793) |
| ^a^The cell count and percentage are presented except in the cases of birthweight percentile for gestational age and median household income, where the mean and standard deviation are provided. ^b^The chi-square test was used to test for differences by race group for categorical variables. The Kruskal-Wallis rank sum test was used birthweight percentile for gestational age and census tract-level median household income. | | | | | |

| **SM Table S6. Odds Ratios by Exposure Period and Race for Small for Gestational Age (SGA) Births per Interquartile Range Increase in Whole-Pregnancy PM_2.5_ (1.9 μg/m^3^) for Sibling Model ^a^** | | | | |
| --- | --- | --- | --- | --- |
| **Non-Hispanic Black women** | **Downscaler [1]**  **(n= 28,782 births to n= 13,797 women)** | **Downscaler [2]**  **(n= 20,264 births to n= 9,675 women)** | **Monitor**  **(n= 20,264 births to n= 9,675 women)** | |
|  | OR  (95% CI) | OR  (95% CI) | OR  (95% CI) | |
| 1^st^ trimester | 1.01  (0.96, 1.06) | 0.96  (0.89, 1.04) | 0.94  (0.87, 1.03) | |
| 2^nd^ trimester | 1.02  (0.97, 1.07) | 1.07  (0.98, 1.15) | 1.07  (0.99, 1.15) | |
| 3^rd^ trimester | 1.05  (1.00, 1.10) | 1.01  (0.94, 1.09) | 0.96  (0.89, 1.03) | |
| Whole pregnancy | 1.04  (0.98, 1.11) | 1.03  (0.92, 1.14) | 0.98  (0.88, 1.09) | |
| **Non-Hispanic White women** | **Downscaler [1]**  **(n= 81,147 births to n=39,617 women)** | **Downscaler [2]**  **(n= 53,286 births to n= 25,953 women)** | **Monitor [2]**  **(n= 53,286 births to n= 25,953 women)** | |
|  | OR  (95% CI) | OR  (95% CI) | | OR  (95% CI) |
| 1^st^ trimester | **1.08**  **(1.03, 1.12)*** | 1.05  (0.96, 1.14) | | 1.07  (0.99,1.16) |
| 2^nd^ trimester | **1.06**  **(1.02, 1.10)*** | 1.02  (0.94, 1.11) | | 1.05  (0.97, 1.14) |
| 3^rd^ trimester | **1.06**  **(1.02, 1.11)*** | 1.04  (0.96, 1.13) | | 1.04  (0.97, 1.13) |
| Whole pregnancy | **1.11**  **(1.06, 1.18)*** | 1.08  (0.96, 1.22) | | **1.13***  **(1.01, 1.26)** |
| Abbreviations: CI, confidence interval; OR, odds ratio; PM_2.5_, particulate matter with aerodynamic diameter of < 2.5 μm.  ^a^ Adjusted for birth order, infant sex, maternal age, maternal educational attainment, maternal smoking during pregnancy, maternal marital status, mean temperature during the corresponding exposure window, and median household income of the census tract of residence.  *Indicates statistical significance at p<0.05. | | | | |

| **SM Table S7. Coefficients by Exposure Period and Race for Birthweight Percentile for Gestational Age per Interquartile Range Increase in Whole-Pregnancy PM_2.5_ (1.9 μg/m^3^) for Sibling Model** | | | |
| --- | --- | --- | --- |
| **Non-Hispanic Black women** | **Downscaler [1] ^a^**  **(n= 28,782 births to n= 13,797 women)** | **Downscaler [2] ^a^**  **(n= 20,264 births to**  **n= 9,675 women)** | **Monitor ^a^**  **(n= 20,264 births to**  **n= 9,675 women)** |
|  | Coefficient  (95% CI) | Coefficient  (95% CI) | Coefficient  (95% CI) |
| 1^st^ trimester | 0.094 (-0.26, 0.45) | 0.19 (-0.27, 0.64) | 0.23 (-0.21, 0.67) |
| 2^nd^ trimester | 0.011 (-0.33, 0.35) | -0.016 (-0.46, 0.42) | -0.084 (-0.51, 0.34) |
| 3^rd^ trimester | -0.16 (-0.49, 0.18) | -0.039 (-0.47, 0.39) | 0.029 (-0.39, 0.44) |
| Whole pregnancy | -0.065 (-0.54, 0.41) | 0.017 (-0.63, 0.67) | 0.069 (-0.57, 0.71) |
| **Non-Hispanic White women** | **Downscaler [1] ^a^**  **(n=81,147 births to**  **n= 39,617 women)** | **Downscaler [2] ^a^**  **(n= 25,953 births to**  **n= 53,286 women)** | **Monitor [2] ^a^**  **(n= 25,953 births to**  **n= 53,286 women)** |
|  | Coefficient  (95% CI) | Coefficient  (95% CI) | Coefficient  (95% CI) |
| 1^st^ trimester | -0.038 (-0.25, 0.17) | 0.11 (-0.19, 0.41) | -0.001 (-0.27, 0.27) |
| 2^nd^ trimester | **-0.34 (-0.55, -0.14)** | -0.15 (-0.44, 0.14) | **-0.35 (-0.61, -0.086)** |
| 3^rd^ trimester | **-0.31 (-0.51, -0.11)** | -0.27 (-0.55, 0.015) | -0.16 (-0.42, 0.089) |
| Whole pregnancy | **-0.46 (-0.74, -0.17)** | -0.31 (-0.76, 0.14) | **-0.48 (-0.88, -0.077)** |
| Abbreviations: CI, confidence interval; OR, odds ratio; PM_2.5_, particulate matter with aerodynamic diameter of < 2.5 μm.  ^a^ Adjusted for birth order, infant sex, maternal age, maternal educational attainment, maternal smoking during pregnancy, maternal marital status, mean temperature during the corresponding exposure window, and median household income of the census tract of residence.  *Indicates statistical significance at p<0.05. | | | |

**SM FIGURES**

| **SM Figure S1. Distribution of within-woman differences in PM_2.5_ concentration among first and subsequent births, by maternal race/ethnicity and exposure window** | |
| --- | --- |
| First trimester | Second trimester |
| 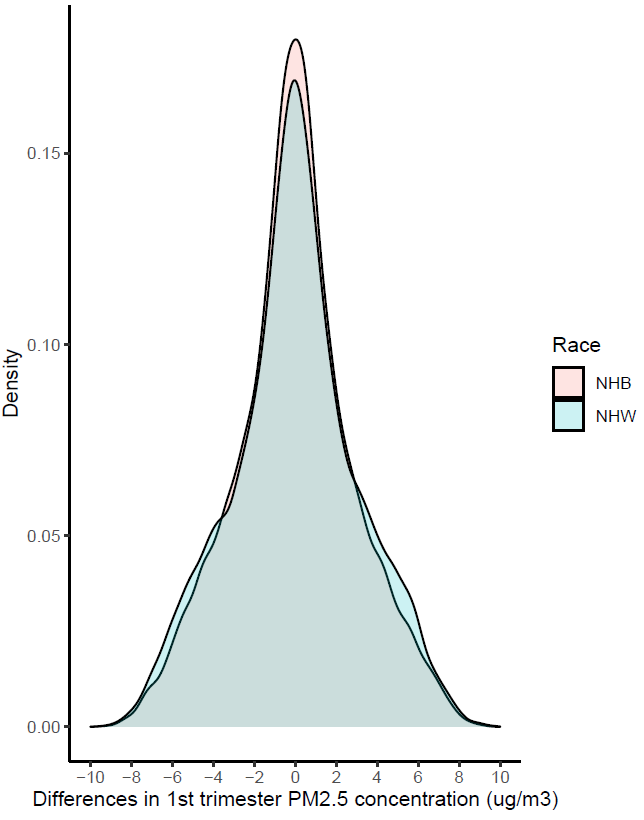 | 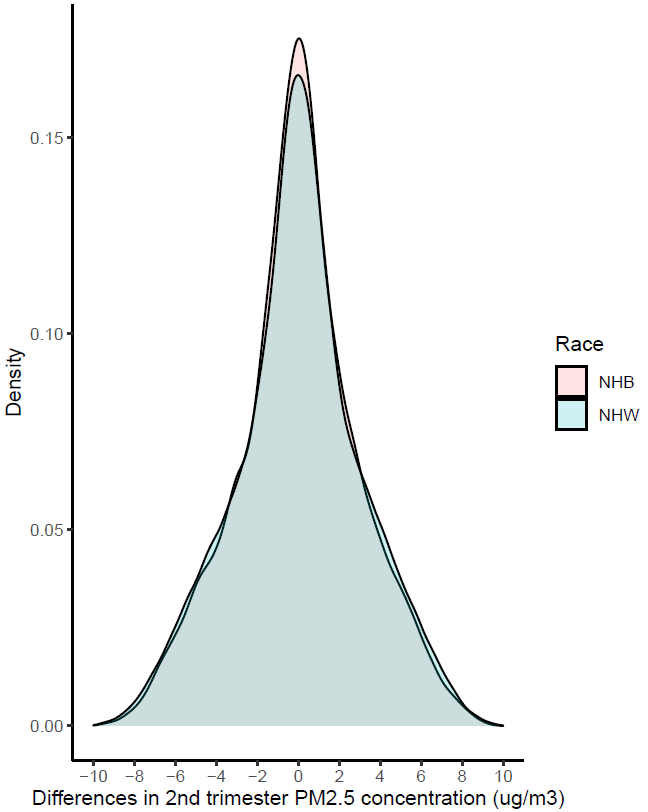 |
| Third trimester | Pregnancy-long |
| 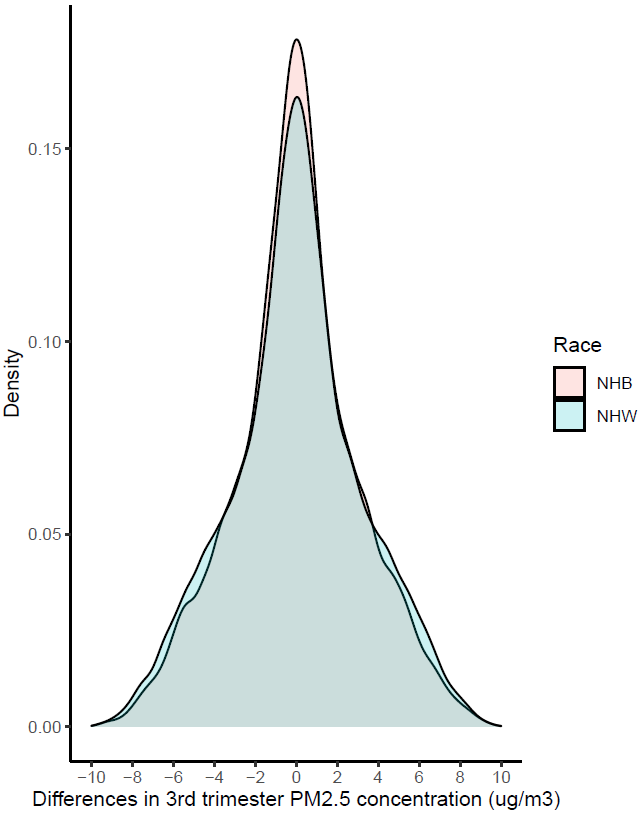 | 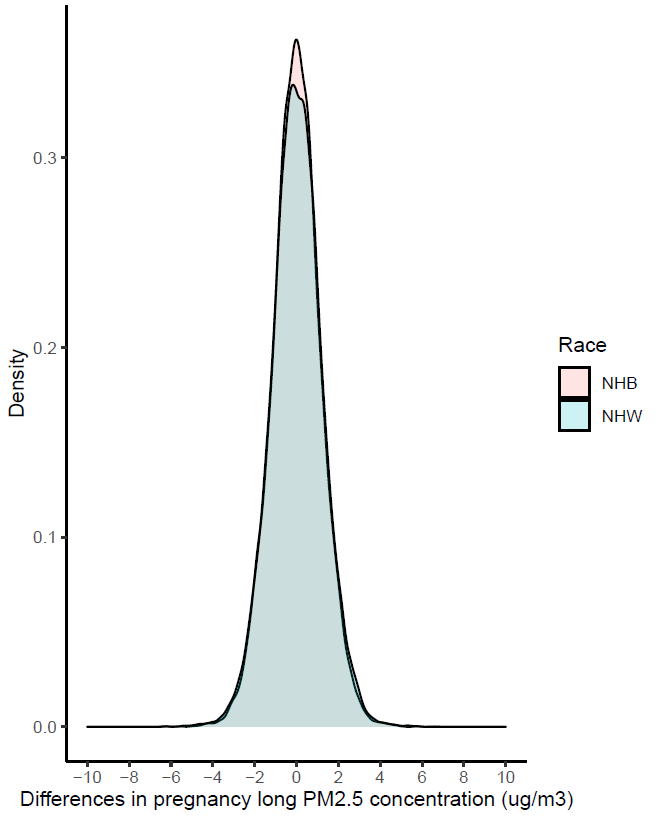 |

| **SM Figure 2. Smoothed associations of trimester-specific and pregnancy-long PM_2.5_ with birthweight percentile for gestational age among non-Hispanic Black mothers** | |
| --- | --- |
| 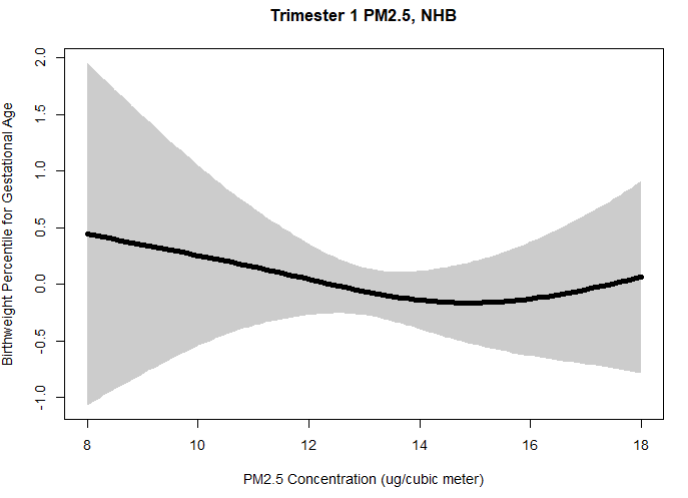 | 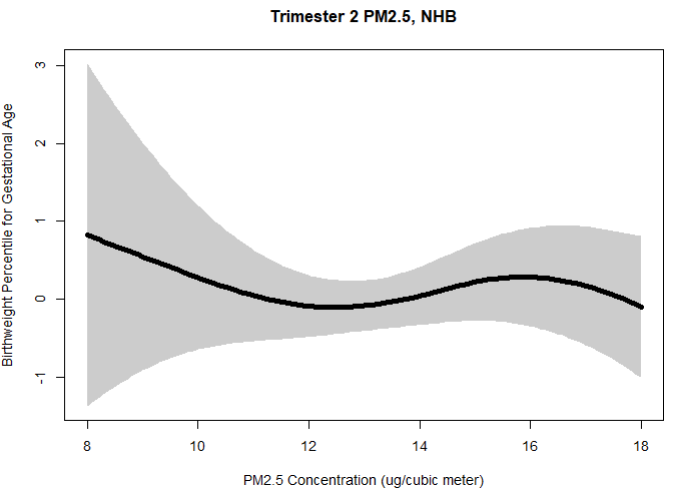 |
| 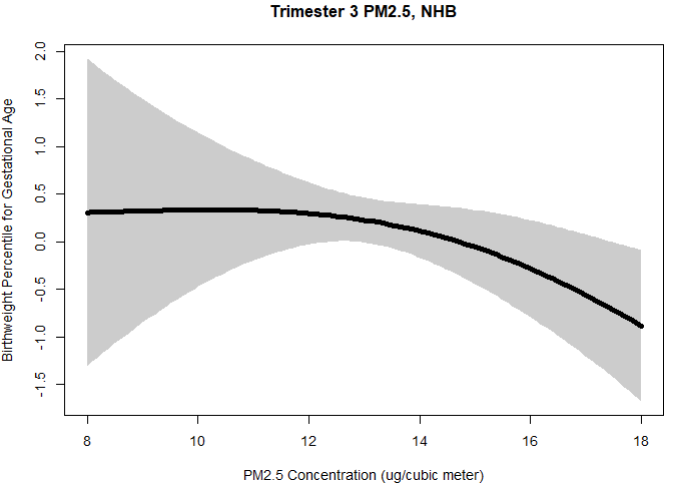 | 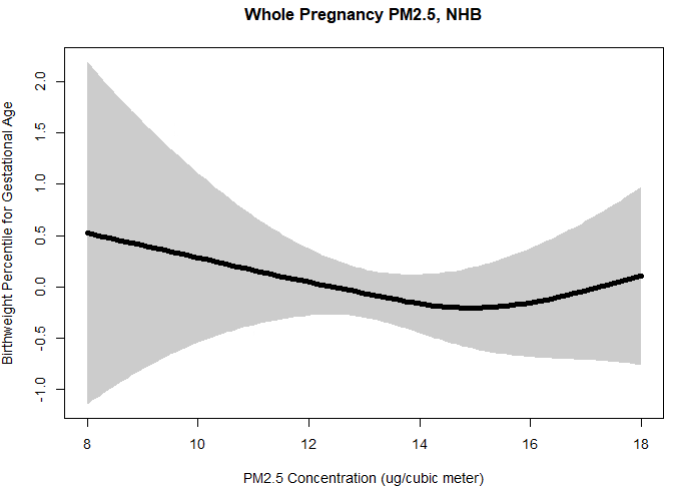 |
|  |  |

| **SM Figure 3. Smoothed associations of trimester-specific and pregnancy-long PM_2.5_ with birthweight percentile for gestational age among non-Hispanic White mothers** | |
| --- | --- |
| 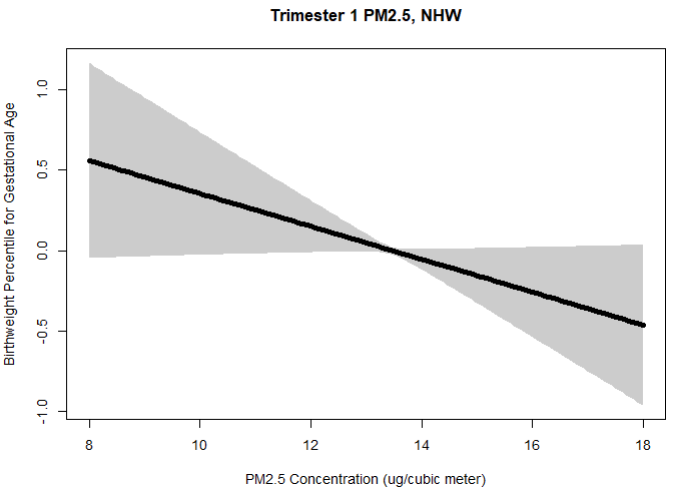 | 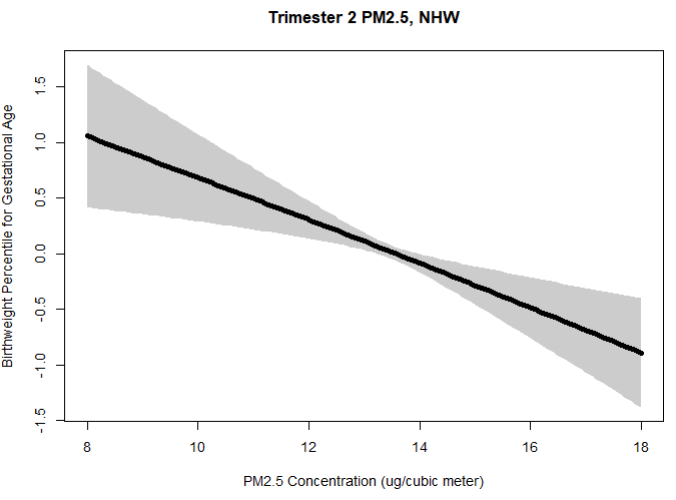 |
| 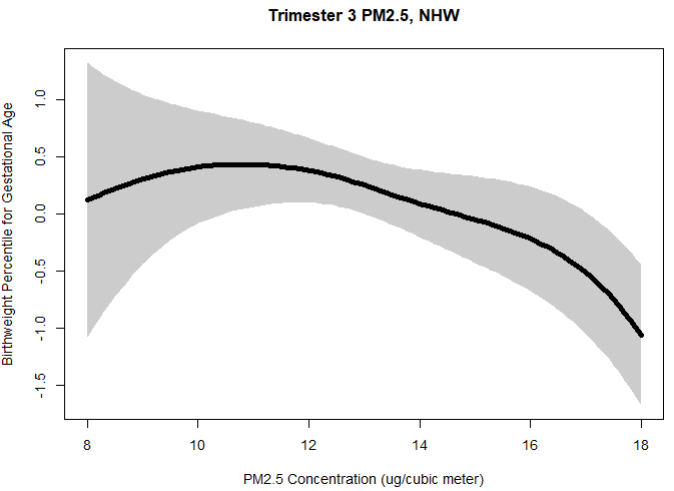 | 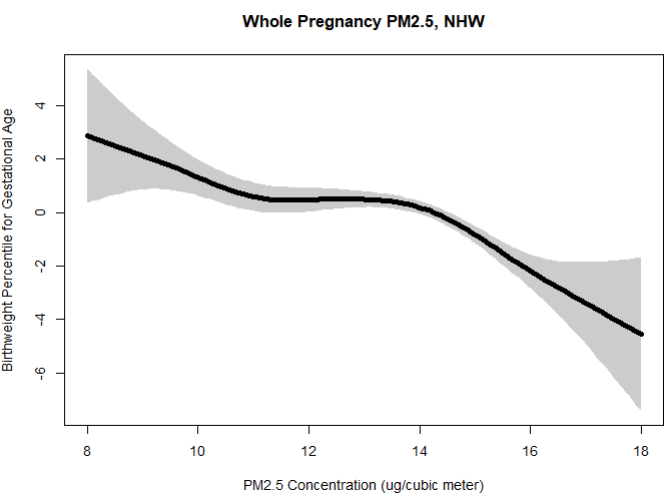 |
|  |  |
